# Hippocampus-Anterior Hypothalamic Circuit Modulates Stress-Induced Endocrine and Behavioral Response

**DOI:** 10.3389/fncir.2022.894722

**Published:** 2022-06-20

**Authors:** Jee Yoon Bang, Julie Zhao, Mouly Rahman, Sophie St-Cyr, Patrick O. McGowan, Jun Chul Kim

**Affiliations:** ^1^Department of Cell & Systems Biology, University of Toronto, Toronto, ON, Canada; ^2^Department of Psychology, University of Toronto, Toronto, ON, Canada; ^3^Department of Cell & Systems Biology, University of Toronto Scarborough, Toronto, ON, Canada

**Keywords:** hippocampus, hypothalamus, stress, hypothalamic - pituitary- adrenal axis, neural circuits, behavior & cognition

## Abstract

Hippocampal input to the hypothalamus is known to be critically involved in mediating the negative feedback inhibition of stress response. However, the underlying neural circuitry has not been fully elucidated. Using a combination of rabies tracing, pathway-specific optogenetic inhibition, and cell-type specific synaptic silencing, the present study examined the role of hippocampal input to the hypothalamus in modulating neuroendocrine and behavioral responses to stress in mice. Transsynaptic rabies tracing revealed that the ventral hippocampus (vHPC) is monosynaptically connected to inhibitory cells in the anterior hypothalamic nucleus (AHN-GABA cells). Optogenetic inhibition of the vHPC→AHN pathway during a restraint stress resulted in a prolonged and exaggerated release of corticosterone, accompanied by an increase in stress-induced anxiety behaviors. Consistently, tetanus toxin-mediated synaptic inhibition in AHN-GABA cells produced a remarkably similar effect on the corticosterone release profile, corroborating the role of HPC→AHN pathway in mediating the hippocampal control of stress responses. Lastly, we found that chronic inhibition of AHN-GABA cells leads to cognitive impairments in both object and social recognition memory. Together, our data present a novel hypothalamic circuit for the modulation of adaptive stress responses, the dysfunction of which has been implicated in various affective disorders.

## Introduction

Stress is broadly defined as a real or perceived threat that disrupts an organism’s well-being or homeostasis (Chrousos, 1992, 2009; Chrousos and Gold, 1992; Ursin and Eriksen, 2004; Koolhaas et al., 2011). In response to stress, various hypothalamic nuclei including the paraventricular nucleus of the hypothalamus (PVN) integrate information from multiple sensory modalities as well as limbic forebrain areas (McEwen et al., 1968; Antoni, 1986; Herman et al., 2003; Myers et al., 2014), which ultimately activate neuroendocrine, autonomic, and behavioral systems to adapt quickly to environmental threats and defend homeostasis. While the adaptive stress response is essential for survival, exaggerated and prolonged stress responses produce deleterious effects, requiring it to be tightly controlled within tolerable limits (Carroll et al., 1976; Whitnall, 1993; Sapolsky, 1996; Soares et al., 2012; Herman et al., 2016). Thus, disrupted control of stress adaptive responses represents a severe threat to the health of organisms and has been widely implicated in the pathophysiology of stress-related psychiatric disorders, especially those involving anxiety and depression (Dunner et al., 1979; Kessler, 1997; Baum and Posluszny, 1999; McEwen, 2008).

In addition to its well-established role in spatial navigation, learning, and memory, the hippocampus has long been thought to play an important role in dampening stress responses (Sapolsky et al., 1984; Herman et al., 1998, 1999; Jacobson and Sapolsky, 1991; Cullinan et al., 1993; Fanselow and Dong, 2010). Previous studies showed that ventral hippocampal (vHPC) lesions encompassing the ventral subiculum and CA1 produce exaggerated neuroendocrine responses after psychological stress (e.g., restraint stress) and upregulate corticotropin-releasing factor (CRF) mRNA expression in the PVN (Herman et al., 1998; Radley and Sawchenko, 2011). Conversely, hippocampal stimulation decreases glucocorticoid secretions in rats and humans (Mandell et al., 1963; Dupont et al., 1972; Dunn and Orr, 1984; Rubin et al., 1996). These data have led to a prevailing view that the vHPC is involved in the negative feedback inhibition of CRF release from the PVN (Sapolsky et al., 1984; Herman et al., 1996, 1998, 2003, 2005; Herman and Mueller, 2006).

Hippocampal inputs to the hypothalamus arise mainly from the subiculum and CA1 regions of vHPC (Kishi et al., 2000; Cenquizca and Swanson, 2006, 2007). Importantly, however, these vHPC neurons do not directly innervate the PVN, instead, they send glutamatergic projections to various PVN-projecting GABAergic structures within and outside the hypothalamic region, including the BNST, medial preoptic area, anterior hypothalamic nuclei, and dorsomedial hypothalamus. These PVN-projecting GABAergic structures indeed show marked c-fos induction in response to stressors (Herman et al., 2005; Radley et al., 2009). Accordingly, it has been proposed that excitatory vHPC signals activate intermediary GABAergic structures interconnecting the vHPC and PVN, enhancing GABAergic tone at the PVN in response to stressors (Sapolsky et al., 1984; Herman et al., 1996, 1998, 2003, 2005; Herman and Mueller, 2006).

Despite the extensive evidence in support of hippocampal control of stress responses, our understanding of the underlying neural circuit is very limited. In particular, it remains unknown which intermediary GABAergic structure is most relevant to the hippocampal inhibition of stress response and whether hippocampal inputs can be selectively manipulated to change physiological and behavioral reactivity to stress. The anterior hypothalamic nucleus (AHN) is a prominently GABAergic structure located lateral to the PVN (Boudaba et al., 1996; Ziegler et al., 2002) and displays a high level of c-Fos expression after exposure to a stressor (Deacon, 2013; Anthony et al., 2014). It receives strong hi ppocampal innervations from the ventral subiculum and CA1 (Kishi et al., 2000; Cenquizca and Swanson, 2006, 2007), and provides direct inhibitory inputs to the CRF-expressing parvocellular neurons in the PVN (Anthony et al., 2014). Together, these findings raise a possibility that the AHN may be an ideal intermediary GABAergic relay structure mediating the hippocampal inhibition of stress responses.

The present study is aimed at elucidating the neural circuit underlying hippocampal inhibition of stress response. We present a series of neural circuit tracing and manipulation experiments targeting the hippocampal-hypothalamic connection. Our data demonstrate that the vHPC inputs to the AHN (i.e., vHPC→AHN pathway) play a critical role in shaping a normal endocrine and behavioral response to stress.

## Methods

### Animals

Adult male mice C57BL/6 (Charles River, USA; *N* = 26) and GAD65-Cre (Gad2tm2(cre) Zjh/J. Strain #010802; *N* = 58) at 8–12 weeks of age were used throughout the study. Mice were group-housed with ad libitum access to food and water in a temperature-controlled room on a 12/12 h light/dark cycle. 10 days prior to behavior testing, mice were switched to a single housing. All experimental procedures were in accordance with the guidelines of the Canadian Council on Animal Care and the local Animal Care Committee at University of Toronto.

### Viral Vectors and Stereotaxic Surgery

AAV2/5-CaMKIIa-eArchT3.0-eYFP, AAV2/8-hsyn-eGFP, and AAV2/5-CaMKIIa-eGFP were purchased from the Addgene and used for optogenetic experiments. AAV2/8-CBA-FLEX-TeLC (a gift from Peer Wulff; Murray et al., 2011) and AAV2/9-CAG-FLEX-eYFP (UNC Vector Core, USA) were used in TOX-mediated synaptic inhibition experiments. EnvA-ΔG-mCherrypseudo typed rabies were purchased from the Salk Institute Vector core facility. For all surgical procedures, mice were anesthetized with isoflurane (4% for induction and 2% for maintenance of anesthesia) at an oxygen flow rate of 1 L/min, and head fixed in a stereotactic frame (David Kopf, USA). The eyes were lubricated with an ophthalmic ointment throughout the surgeries. Ketoprofen was provided for pain management during post-operative recovery. For the ventral hippocampus/subiculum infusion (AP -3.8mm, ML -2.1 mm, DV -4.8 mm, 10° away from the midline), 300 nl per site were infused by cannula needle connected to Tygon tubing to a 10-μl Hamilton syringe (Hamilton Company, USA) at rate 0.1 μl/ min. Custom-made ferrule fibers consisting of optic fibers (200 μm core diameter, 0.39 NA, Thorlabs) threaded in 1.25 mm wide zirconia ferrules (Thorlabs) were implanted at the AHN (AP -0.85 mm, ML 1.38 mm, DV -5.1 mm, 10° towards the midline). For the AHN infusion (AP -0.85 mm, ML 0.45 mm, DV -5.2 mm), 69 nl per site was infused by a pulled glass needle and Nanoject II (Drummond Scientific, USA) at 46 nl/s rate, and the needle was left in place for additional 10 min to limit the virus drag during needle retract. Coordinates were selected based on Paxinos and Franklin atlas. Post-surgery viral transduction time was minimum 2 weeks for the optogenetic experiments and 3 weeks for TOX-mediated synaptic inhibition experiments. For cell type specific monosynaptic retrograde tracing, GAD65-Cre mice received an injection of AAV-FLEX-TC66T-eGFP-2A-oG (41.4 69 μl) in the AHN with Nanoject II. After 2 weeks of AAV transfection, EnvA-ΔG-mCherry pseudo typed rabies was injected to the same coordinate.

### Stress-Induced Serum Corticosterone Quantification

Age-matched male mice (HPC-AHN inhibition: GFP *N* = 5, ArchT *N* = 7; AHN GABA synaptic function loss: GFP *N* = 5, TOX *N* = 5) were subjected to an acute stress for 30 min in a physical restraint (DecapiCone, Braintree Scientific, USA). The DecapiCone was cut out to accommodate the connection of optic cable on animals’ heads when necessary. The blood sampling was carried out from 11 a.m. to 3 p.m. to control for circadian rhythm induced variation of circulating corticosterone (Malisch et al., 2008). A small nick on the tail vein allowed the sampling of 20 μl blood at multiple timepoints per animal before, during and after a 30 min physical restraint. After physical restraint, mice were returned to their home cage and gently retrieved for additional blood collection. Blood samples were centrifuged at 4°C at 4,000 rpm for 20 min, and serum was collected and stored at −80°C. Serum corticosterone levels were measured using the Corticosterone Double Antibody RIA Kit with 125I-labeled anti-corticosterone antibody (MP biomedical, USA, cat. 07120103; sensitivity 7.7 ng/ml, intra-assay coefficient of variation 0.48%, inter-assay coefficient of variation 0.69%). The samples were run in duplicate in a 1:200 dilution. CORT concentrations in samples were calculated from a standard curve with eight points increasing in 1:2 increments ranging from 6.25 to 1,000 ng/ml. Integrated CORT is reported as the value calculated by the area under the curve across the time points.

### Anxiety-Related Behavior Tests

Open field (OF, 20 min) and successive alleys (SUA, 5 min) tests were conducted in a counterbalanced order in mice after acute physical restraint stress (30 min) or in stress-naïve mice (HPC-AHN inhibition: GFP *N* = 7, ArchT *N* = 7, AHN GABA synaptic function loss: GFP *N* = 13, TOX *N* = 10 12). Between behavior tests, mice were placed back into their home cages for 2–5 min, which allows for assessing the prolonged state of anxiety after acute physical restraint stress (Anthony et al., 2014). Behavior tracking was carried out by ANY-MAZE software (Stoelting Co, USA). The OF apparatus was a clear plexiglass chamber (50 cm × 50 cm × 20 cm). The SUA apparatus consisted of elevated four linearly connected alleys (alley 1–4) with increasing anxiogenic features (McHugh et al., 2004; Deacon, 2013). The first alley (alley 1) is enclosed and painted in black and is followed by three lighter colored open alleys (Alley 2: gray, Alley 3 and 4: white) where the width of the four alleys progressively decreases.

### Novel Object Recognition Test (NORT)

Short-term object recognition memory was assessed using the novel object recognition test (NORT) with a 30-min delay (GFP *N* = 8, TOX *N* = 9). The NORT consisted of habituation (10 min), object encoding (10 min), delay period (30 min), and object discrimination phase (10 min). Mice were first introduced to a chamber (30 cm W × 30 cm L × 30 cm H) to freely roam for a 10 min habituation. During the object encoding, two identical objects (O1 and O2, 14 cm apart and aligned) were introduced. During the 30-min delay, mice were placed back in the home cage. A copy of the previously exposed object (O3) and a novel object (NO) were presented for 10 min in the same testing chamber. The object interaction was scored manually in a treatment blinded manner with the assistance of the ANY-MAZE software. Interaction with objects was detected as direct nose, paws, or mouth contact or approaching towards the object within 1 cm in distance. Sitting or climbing on the object did not count as an interaction. Between each trial, the chamber and objects were thoroughly cleaned with 70% ethanol and deionized water to remove any odor cue. The time spent exploring each object was calculated as a fraction of the total exploration time for each trial. The discrimination index was calculated as the [NO exploration time − O3 exploration time)/total exploration time].

### Three-Chamber Sociability and Social Novelty Test

Sociability and social novelty tests were performed in a clear Plexiglass three-chambered box (45 cm W × 20 cm L × 30 cm H) with removable partitions between the chambers (GFP *N* = 11, TOX *N* = 8). The left and the right chambers had identical cylindrical wire cages (8 cm diameter, 17 cm H) with bars (1 cm apart) suitable for holding stranger conspecifics. The three-chamber test consisted of a habituation phase (10 min) to the empty apparatus, sociability phase (5 min), delay phase (3 min), and social recognition phase (5 min). Between the start of each test phase, test subjects were enclosed in the empty center chamber. During the habituation phase, animals were allowed to freely roam all three chambers. During the sociability phase, a juvenile mouse (4–7 weeks old) of the same sex was introduced in one side of the chamber, while the other side remained empty. Anogenital sniffing, grooming, and nose pokes into the cylindrical cage directed at the stranger juvenile mouse were quantified as social interaction. Climbing on the wire cage did not count as social interaction. The manual behavior scoring was carried out in a treatment blind manner with the assistance of the ANY-MAZE. In the social novelty phase, a novel juvenile mouse was placed in the previously empty wire cage. Social novelty preference was calculated as a discrimination index: [(Novel mouse social investigation time − Familiar mouse social investigation time)/Total time of social investigation].

### Optogenetic Apparatus

Optogenetic inhibition of hippocampal terminals expressing ArchT-GFP in the AHN was achieved by a continuous illumination with green light (532 nm, 12 mW) from a diode-pumped solid-state laser (Laserglow, Toronto, ON, Canada). The laser was connected to a 1 × 2 optical commutator (Doric Lenses, Quebec, QC, Canada), which divided the light path into two arena patch cables attached to the bilaterally implanted optical fibers.

### Histology and Microscopy

Mice were anesthetized with avertin and underwent transcardial perfusion with 0.1 M phosphate-buffered saline (PBS, pH 7.4), followed by 4% paraformaldehyde (PFA). The brain tissues were carefully removed into ice-cold 4% PFA for overnight tissue fixation. Additional 48 h of incubation in 30% sucrose solution followed for cryoprotection for brain tissues. Free-floating coronal (40 μm) were cut with a cryostat (Leica, Germany), permeabilized with PBS containing 0.3% Triton X-100 (PBS-T), and blocked with 5% normal donkey serum (Jackson ImmunoResearch, USA). Tissue sections were then incubated with PBS-T containing primary antibodies for 48 h at 4°C, followed by AlexaFluor 488- or 594-conjugated donkey secondary antibodies (1:500 in PBS-T, Jackson ImmunoResearch, USA) for 2 h at room temperature, and slide-mounted with Aquamount (Polysciences, Inc., Warrington, PA). For visualization of TOX expression spread in the anterior hypothalamus, GFP antibody (chicken anti-GFP, Abcam, ab13970, 1:1,000 in 0.1% PBS-T) and NeuN (rabbit anti-NeuN, Abcam, ab177487, 1:1,000 in 0.1% PBS-T) was used as primary antibody. To confirm and quantify the efficacy of synaptic functional loss, VAMP2, synaptobrevin antibody (rabbit anti-VAMP2, synaptic systems, 104 202, 1:500, in 0.1% PBS-T) was used as the primary antibody. 20× objective confocal images were taken using equal exposure setting for all mice. Image J was used to detect and quantify overlap of VAMP2 and GFP signals in a treatment-blinded manner. Percent overlap was calculated as the area of colocalization over area of GFP signal in GFP control or TOX-GFP. Wide-field fluorescent microscope images were captured using a 4× objective lens on a fluorescent microscope (Olympus, Japan). Confocal images were captured using a 20 × 40 × 60× objective through a LSM800 confocal microscope (Zeiss, Germany).

### Statistical Analysis

All statistical analysis was performed using GraphPad Prism (GraphPad Software). Significance was determined using a (two-tailed) unpaired Student’s *t*-test (two-tailed), one-sample *t*-test, ordinary one-way analysis of variance (ANOVA), two-way repeated-measures ANOVA. Bonferroni’s multiple comparisons test was used for *post-hoc* comparisons. Significance was defined as *P < 0.05, **P < 0.005, ***P < 0.0005, ****P < 0.0001.

## Result

### AHN GABAergic Neurons Receive Monosynaptic Inputs From the Hippocampal Formation

The AHN has been proposed as an ideal candidate for intermediary GABAergic relay structure that mediates the negative feedback inhibition of stress responses. Thus, we first sought to identify the source of presynaptic inputs arriving at AHN GABA cells using the retrograde-monosynaptic tracing *via* glycoprotein-deleted rabies virus. To target AHN GABA cells for rabies tracing, GAD65-Cre mice were first infused with AAV-FLEX-TC66T-eGFP-2A-oG into the AHN to express envelope A receptor (TVA/TC66T) and rabies glycoprotein (oG). Two weeks after AAV surgery, pseudotyped rabies (EnvA+RVdG-mCherry) was infused into the same region of the AHN and then incubated another 4 days to provide time for retrograde trans-synaptic viral spread. To analyze the labeling density and distribution, starter cells were identified as cells infected by both AAV and rabies virus, determined by co-localization of GFP and mCherry, while mono-synaptically connected presynaptic cells were identified with mCherry.

**Figure 1:**
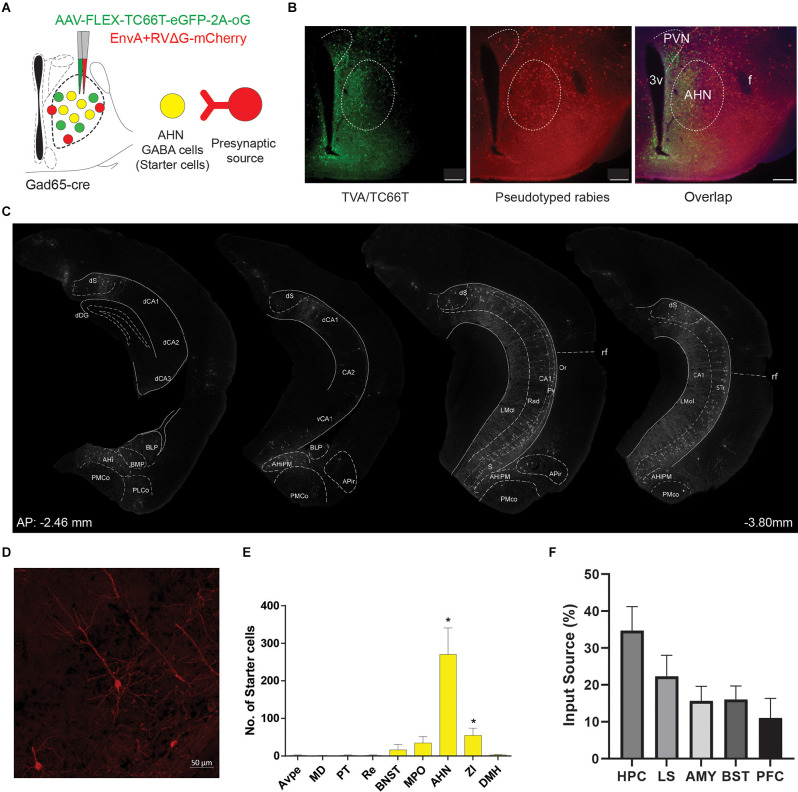
AHN GABA cells receive inputs from the hippocampus. **(A)** Schematic showing the cell type specific monosynaptic tracing approach. AAV-FLEX-TC66T-eGFP-2A-oG (green), cre-dependent helper virus expresses a*via*n viral receptor TVA and rabies G in the anterior hypothalamic GABA cells. 2 weeks later,EnvA-ΔG rabies expressing mcherry infects GABA cells expressing TVA and retrogradely labels one synapse away with the help of rabies G protein. Yellow cells, starter cells express TVA and rabies G and EnvA. Red cells, presynaptic input source that project to the starter cells. **(B)** A representative section showing localized expression of GABAergic starter cells in the anterior hypothalamus (AHN) shown by expression of AAV-FLEX-TC66T-eGFP-2A-oG (green, left), EnvA-RVΔG (mCherry, center), and overlapped expression (green + mCherry, right). Scale bar = 100 μm. **(C)** Schematic showing the presynaptic neuron source in the hippocampus from anterior to posterior axis. **(D)** A representative 40x confocal microscope image of ventral subiculum pyramidal neuron that projects to the AHN GABA cells. **(E)** Quantification of starter cells (*N* = 5). **(F)** Quantification of the presynaptic input source among limbic structures involved in control of HPA-axis (1-WAY ANOVA, 0.2661_(4,20)_ = 0.8963, *p = 0.036, Dunnett’s multiple comparisons test, HPC vs. PFC, *p = 0.0139, HPC vs. LS, *p* = 0.2929, NS, HPC vs. AMY, *p* = 0.0542, NS, HPC vs. BST, *p* = 0.0602, NS). 3v, third ventricle; Ahi, amygdalohippocampal area; APir, amygdalopiriform transition area; AVPe, anteroventral periventrivular nucleus; BLP, basolateral amygdaloid nucleus, posterior part; BMP, basomedial amygdaloid nucleus, posterior; BNST, bed nucleus of stria terminalis; dCA1, dorsal cornu ammonia 1 of hippocampus; dCA2, dorsal cornuammonis 2 of hippocampus; dCA3, dorsal cornuammonis 3 of hippocampus; dDG, dorsal dentate gyrus; DMH, dorsomedial hypothalamus (DM); dS, dorsal subiculum; Lmol, lacunosummoleculare layer of the hippocampus; MD, mediodorsal thalamic nucleus; PLCo, posterolateral cortical amygdaloid area; PMCo, posteromedial cortical amygdaloid area; PVN, paraventricular nucleus; PT; Rad, radiatum layer of the hippocampus; rf, rhinal fissure; STr, subiculum transition area; ZI, zona incerta.

We found that starter cells co-expressing GFP and mCherry were largely restricted within the AHN (Figures 1A,B,E). mCherry-positive, AHN-projecting presynaptic cells were found throughout various distal structures including medial, lateral and central amygdala, ventrolateral portions of the lateral septum, deep layers of prefrontal cortex (IL, PrL), anterior cingulate cortex (Cg1, Cg2), orbitofrontal cortex (MO, VO, LO), insular cortex, bed nucleus of the stria terminalis (BNST), and the hippocampal formation including vCA1, dSub, and vSub. A subsequent quantification revealed that approximately 35% of the AHN GABA cell-projectors of major limbic structures are located in the hippocampus (HPC; Figures 1C,F), where the labeled cells were found exclusively in the pyramidal layers and displayed a typical pyramidal cell morphology (Figure 1D). Within the hypothalamus, presynaptic cells were found in PVN, lateral hypothalamus (LH), posterior hypothalamus (PH), dorsomedial hypothalamus (DM), ventromedial hypothalamus (VMH), dorsal premammillary nucleus (PMD) and medial preoptic area (MPA; data not shown). Thus, our retrograde tracing experiments showed that the AHN GABA cells receive direct monosynaptic inputs from both local hypothalamic neurons and distant brain areas and that the vHPC provides a major source of inputs to the AHN from outside of the hypothalamus.

### HPC→AHN Inputs Are Necessary for Negative Feedback Inhibition of HPA Activity

**Figure 2:**
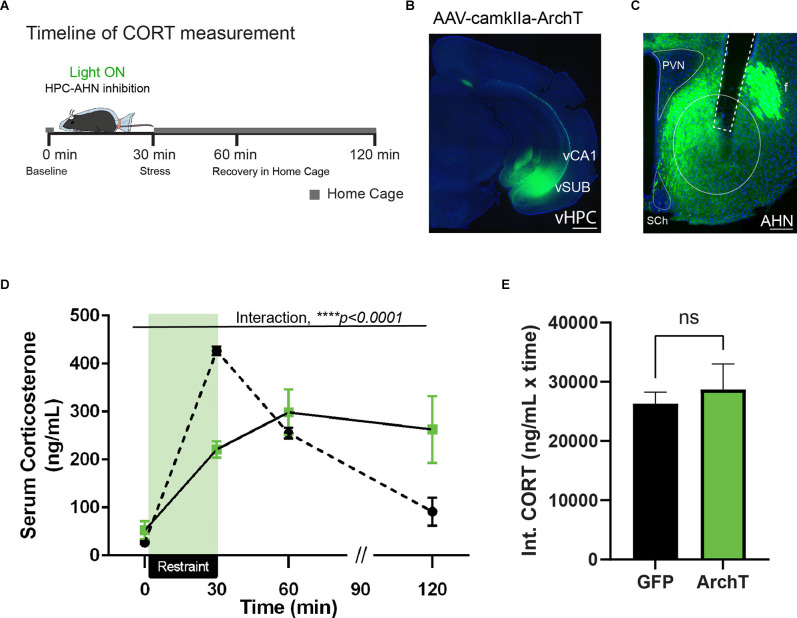
HPC→AHNis necessary for negative feedback of HPA-axis. **(A)** Acartoon schematic of serum CORT measurement relative to physicalrestraint induced stress. **(B)** A representative image ofventral hippocampus with AAV-camkII-ArchT-GFP transfection. Theventral portion of the temporal hippocampus. vCA1, ventralcornuammonis 1; vSUB, ventral subiculum. Scale bar = 500 μm.Green, ArchT expression. **(C)** Ventral hippocampal terminalslabeled in GFP densely innervate the anterior hypothalamus (AHN),and do not innervate the paraventricular nucleus (PVN). Sch,suprachiasmatic nucleus. Scale bar = 100 μm.**(D)** HPC→AHN inhibition during physical restraint stress decreased peak corticosterone release and impaired negative feedback of the HPA-axis (GFP *N* = 5, ArchT *N* = 7, 2-WAY RM ANOVA, time × rhodopsin, *F*_(3,30)_ = 11.43, ****p < 0.0001, time effect, *F*_(1.499,14.99)_ = 30.29, ****p < 0.0001, rhodopsin effect, *F*_(1,10)_ = 0.0188, *p* = 0.8937, NS, Bonferroni’s multiple comparisons test, *t* = 0 GFP vs. ArchT, *p* > 0.9999, *t* = 30 GFP vs. ArchT, ****p < 0.0001, *t* = 60 GFP vs. ArchT, *p* > 0.9999, *t* = 120 GFP vs. ArchT, *p* = 0.2617, NS, Bonferroni’s multiple comparisons test across time, GFP *t* = 0 vs. *t* = 30, ***p < 0.0001, GFP *t* = 0 vs. *t* = 60, ***p = 0.0008, *t* = 0 vs. *t* = 120, *p* = 0.8184, NS, *t* = 30 vs. 60, **p = 0.004, *t* = 30 vs. *t* = 120, **p = 0.0053, *t* = 60 vs. *t* = 120, *p = 0.0343, ArchT *t* = 0 vs. *t* = 30, **p = 0.0014, *t* = 0 vs. *t* = 60, *p = 0.0151, *t* = 0 vs. 120, *p* = 0.0994, NS, *t* = 30 vs. *t* = 60, *p* > 0.9999, NS, *t* = 30 vs *t* = 120, *p* > 0.9999, NS, *t* = 60 vs. 120, *p* > 0.9999). **(E)** Integrated CORT (Int. CORT) reported comparable total circulating CORT concentration between GFP and ArchT mice during stress-induced HPA-axis (unpaired *t*-test, *t* = 0.1606, df = 10, *p* = 0.8756, NS). All results reported are mean ± s.e.m. *p < 0.05, **p < 0.01, ***p < 0.001, ****p < 0.0001.

The effect of the vHPC lesion is most pronounced during the recovery phase of stress-induced glucocorticoid secretion, resulting in exaggerated corticosterone (CORT) release after stress (Herman et al., 1998; Radley and Sawchenko, 2011). Given our retrograde tracing results, we hypothesized that vHPC input to the AHN serves as a key element of neural circuits terminating the prolonged hypothalamic–pituitary–adrenal (HPA) axis activity. To address the role of the vHPC-AHN pathway in shaping stress responses, we optogenetically inhibited vHPC terminal activity at the AHN during a physical restraint and then assessed how the pathway specific inhibition changed a CORT release profile during and after restraint stress (Figure 2A).

Adeno-associated virus (AAV) carrying the inhibitory opsin, ArchT under the control of the excitatory neuron-specific CaMKII promoter was injected bilaterally into the vHPC along with optic fibers implanted over the AHN to illuminate vHPC axon terminals. As a control, AAV expressing only GFP was used. The viral transduction was confirmed to include all hippocampal presynaptic sources of the AHN, including the ventral CA1 and ventral subiculum (Figure 2B). The expression pattern of GFP control animals did not differ significantly (data not shown). GFP-positive axon terminals were detected in the known targets of vHPC, including the amygdala, lateral septum, nucleus accumbens, and prefrontal cortex (data not shown). Within the hypothalamus, vHPC axon terminals were found most abundant in the AHN (Figure 2C). Of note, the PVN was almost completely excluded from the vHPC innervation.

Four to 6 weeks after viral infusion, mice were exposed to an acute 30 min restraint throughout which mice received a constant green light illumination in the AHN, along with repeated blood samplings at time points before and (0 min 30 min 60 min, and 120 min) after stress (Figure 2D). If the vHPC-AHN pathway is normally required for hippocampus-dependent negative feedback inhibition of HPA activity, the pathway inhibition would lead to an exaggerated and prolonged CORT release after the stress is terminated. As expected, GFP control mice displayed a rapid increase in CORT after a 30 min restraint stress that returned to baseline level at 120 min time point (Figure 2D). In contrast, ArchT mice showed a significantly blunted CORT response after a 30 min restraint stress compared to GFP controls. Strikingly, however, CORT level in ArchT mice remained high even at 60 min and 120 min time points, failing to return to baseline level (Figure 2D). Integrated circulating CORT levels induced by restraint stress across the 120 min did not differ between the GFP and ArchT mice (Figure 2E). Thus, our findings show that the vHPC-AHN pathway plays an important role in shaping the normal stress-induced endocrine response.

### Optogenetic Inhibition of vHPC→AHN Pathway During Stress Increases Anxiety Behaviors

Along with the short-term endocrine responses of the HPA axis, stress produces other lasting physiological and behavioral changes that continue even after the stress has passed. In particular, the appropriate control of stress response is crucial for the maintenance of normal anxiety behaviors after stressful events. The prolonged effect of vHPC→AHN pathway inhibition on serum CORT level (Figure 2D) suggests that ArchT mice may also display abnormal behavioral responses to stress. Therefore, we evaluated how the bilateral optogenetic inhibition of vHPC→AHN pathway during stress would later on impact stress-induced anxiety behaviors.

**Figure 3:**
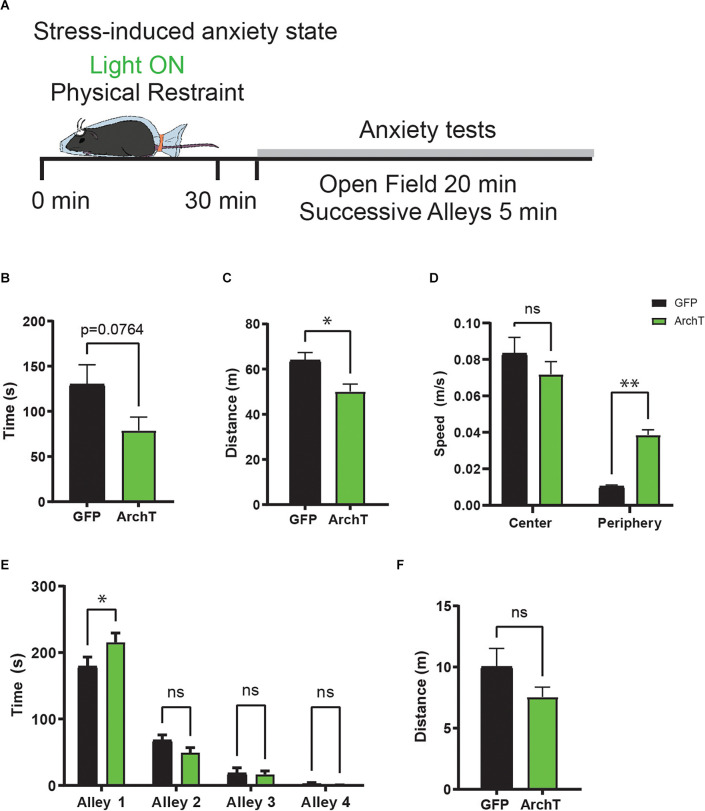
HPC→AHNinhibition during stress increases anxiety behaviors. **(A)** Acartoon schematic of stress-induced anxiety behavior paradigm.**(B)** Time spent in the center of open field shows reducedtrend (GFP *N* = 7, ArchT *N* = 7, unpaired *t*-test, *t* = 1.939, df = 12, *p* = 0.0764, NS). **(C)** Distance traveled in open field (unpaired *t*-test, *t* = 2.924, df = 12, *p = 0.0127). **(D)** Mean speed (m/s) of centerzone did not differ between ArchT and GFP. Mean speed (m/s) of periphery zone was increased in ArchT mice (2-WAY ANOVA, Zone × rhodopsin, *F*_(1,24)_ = 11.66, **p = 0.0023, zone effect, *F*_(1,24)_ = 83.45, ****p < 0.0001, rhodopsin effect, *F*_(1,24)_ = 2.121, *p* = 0.1582, NS, Bonferroni’s multiple comparisons test, CenterArchT vs. GFP, *p* = 0.358, NS, Periphery ArchT vs. GFP, **p = 0.0042). **(E)** ArchT mice prefer closed alley (alley 1) and avoid open alleys (alley 2–4) compared to GFP (2-WAY ANOVA, Alley × rhodopsin effect, *F*_(3,48)_ = 3.524, *p = 0.0218, Alley effect, *F*_(3,48)_ = 207.1, ****p = < 0.0001, rhodopsin effect, *F*_(1,48)_ = 0.2777, *p* = 0.6007, NS, Bonferroni’s multiple comparisons test, Alley 1 GFP vs. ArchT, *p* = 0.0221, Alley 2 GFP vs. ArchT, *p* = 0.5252, NS, Alley 3 GFP vs. ArchT, *p* > 0.9999, NS, Alley 4 GFP vs. ArchT, *p* > 0.9999, NS). **(F)** Distance traveled in successive alleys test (unpaired *t*-test, *t* = 1.449, df = 12, *p* = 0.1731, NS). All results reported are mean ± s.e.m. *p < 0.05, **p < 0.01, ***p < 0.001, ****p < 0.0001.

After the restraint stress procedure, mice were transferred to a different room and tested for anxiety-related behaviors in two rodent behavioral models: open field test (OF) and successive alleys test (SUA; Figure 3A). The OF test measures general locomotor activity levels as well as anxiety-related behaviors (e.g., duration of time spent in the center). The SUA apparatus consists of four successive linearly connected alleys (alley 1–4; McHugh et al., 2004; Deacon, 2013). The first alley (alley 1) is enclosed and painted in black and is followed by three light colored open alleys (alley 2–4). The width of the four alleys progressively decreases to increase their anxiogenic character; therefore, anxious mice tend to avoid the open alleys (alley 2–4). In the OF test, ArchT mice exhibited reduced overall locomotion compared to the GFP with a trend of less time in the center compared to GFP mice (Figures 3B,C). Notably, despite the reduced locomotor activity, ArchT mice exhibited significantly increased speed in the periphery compared to the GFP mice (Figure 3D). In the SUA test, ArchT mice showed significantly greater time spent in the enclosed alley 1 compared to GFP mice, reflecting an increase in anxiety-related behaviors (Figures 3E,F). Together, these data suggest that inhibiting the vHPC→AHN pathway during restraint stress leads to an increase in stress-induced anxiety behaviors.

### Inhibition of AHN GABA Cells Disrupts Negative Feedback Inhibition of HPA Activity

**Figure 4:**
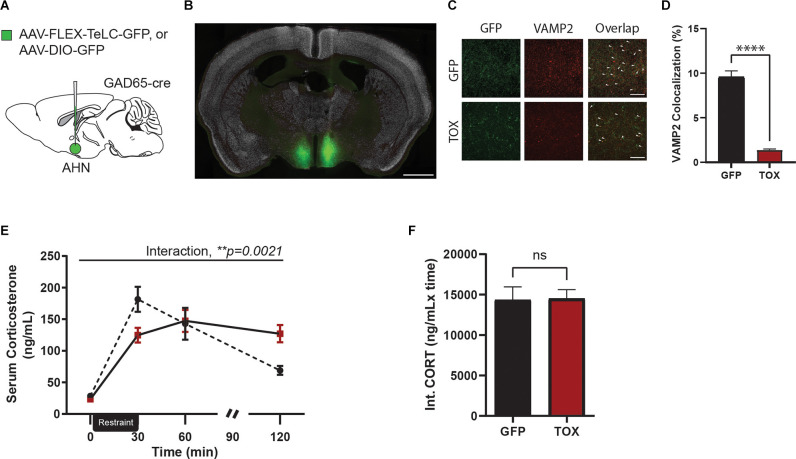
AHN GABAergic synaptic functional loss induced disruption of negative feedback of HPA-axis. **(A)** A cartoon diagram demonstratingexperimental approach to AHN GABA synaptic function loss. **(B)** A representative image of AHN with AAV-FLEX-TeLC-GFP. Green, TOX. White, NeuN. Scale bar = 1 mm. **(C)** Comparison of Vamp2 expression in AHN GABA synaptic terminals, 20x confocal microscope images in GFP (first row) and TOX (second row) mice. Left, GFP positive AHN GABA synaptic terminals. Center, Vamp2 expression. Right, an overlap of GFP and VAMP2 expression. White arrow denotes signal colocalization. Scale bar = 20 μm. **(D)** VAMP2 and GFP colocalization (%) was significantly reduced in synaptic terminals of TOX treatment compared to those of the GFP (GFP *N* = 5, TOX *N* = 5, unpaired *t*-test, *t* = 12.05, df = 157, ****p < 0.0001). **(E)** Chronic inhibition of AHN GABAergic synapses results in impaired peak CORT release and led to prolonged CORT exposure in TOX mice but not in GFP mice (2-WAY RM ANOVA, time × AHN treatment, *F*_(3,30)_ = 6.189, **p = 0.0021, time effect, *F*_(3,30)_ = 38.05, ****p < 0001, AHN treatment effect, *F*_(1,10)_ = 0.000209, *p* = 0.9888, NS, Bonferroni’s multiple comparisons test, *t* = 0 GFP vs. TOX, *p* > 0.9999, NS, *t* = 30 GFP vs. TOX, *p = 0.0314, *t* = 60 GFP vs. TOX, *p* > 0.9999, *t* = 120 GFP vs. TOX, *p = 0.0266, Bonferroni’s multiple comparisons test across time, GFP *t* = 0 vs. *t* = 30, ****p < 0.0001, GFP *t* = 0 vs. *t* = 60, ****p < 0.0001, *t* = 0 vs. *t* = 120, *p* = 0.3427, NS, *t* = 30 vs. 60, *p* = 0.4102, NS, *t* = 30 vs. *t* = 120, ****p < 0.0001, *t* = 60 vs. *t* = 120, **p = 0.0065, TOX *t* = 0 vs. *t* = 30, ****p < 0.0001, *t* = 0 vs. *t* = 60, ****p < 0.0001, *t* = 0 vs. 120, ****p < 0.0001, *t* = 30 vs. *t* = 60, *p* > 0.9999, NS, *t* = 30 vs *t* = 120, *p* > 0.9999, NS, *t* = 60 vs. 120, *p* > 0.9999, NS). **(F)** Integrated CORT (Int. CORT) reported comparable total circulating CORT concentration between GFP and TOX mice during stress-induced HPA-axis (unpaired *t*-test, *t* = 0.08955, df = 10, *p* = 0.9304, NS). All results reported are mean ± s.e.m. *p < 0.05, **p < 0.01, ***p < 0.001, ****p < 0.0001.

Our findings thus far have established a monosynaptic connection between vHPC and AHN GABA cells and shown an important role for the HPC→AHN pathway activity in shaping normal endocrine and behavioral responses to stress. However, it remains to be corroborated whether AHN-GABA cells are directly involved in controlling stress responses. To this end, we investigated the role of AHN GABA cells in regulating stress response by blocking synaptic transmission from AHN GABA cells using tetanus toxin light chain (TOX). AHN-GABA cells were virally transduced for expression of TOX by bilaterally infusing inhibitory neuron-specific GAD65-Cre mice with Cre-responsive AAV-FLEX-expressing GFP-fused TOX (TOX mice) or GFP (GFP control; Figure 4A), resulting in GFP-TOX expression largely restricted within the AHN (Figure 4B). Once expressed in neurons, TOX efficiently blocks synaptic transmission by cleaving VAMP2, a synaptic vesicle protein required for synaptic vesicle fusion (Schiavo et al., 2000). As expected, VAMP2 immunoreactivity in GFP-positive neural processes in the AHN was reduced by 7-fold in TOX mice compared to GFP control mice (Figures 4C,D), suggesting that TOX efficiently cleaved VAMP2 in AHN GABA cells.

Four to 6 weeks after viral infusion, mice (GFP or TOX group) were exposed to an acute 30 min restraint procedure along with repeated blood samplings at time points before and (0 min 30 min 60 min 90 min, and 120 min) after stress (Figure 4D). While there was no change in baseline CORT level, TOX mice displayed a remarkably similar CORT profile to ArchT mice that was observed in the HPC→AHN pathway inhibition experiment (Figure 4E). GFP control mice reached a CORT peak at 30 min time point and returned to baseline level within 120 min. In contrast, TOX mice showed a blunted initial CORT response at 30 min time point, reached its peak at 60 min time point, and continued to maintain a near peak CORT level at both 90 min and 120 min time points. Furthermore, the integrated circulating CORT levels induced by restraint stress across the 120 min did not differ between the GFP and TOX mice (Figure 4F). Thus, our findings showed that AHN GABA cells play an important role in controlling stress-induced endocrine responses.

### Chronic Inhibition of AHN GABA Cells Leads to Dynamic Impairments in Autonomic, Affective, and Cognitive Impairments

In contrast to short-term reversible effects of an acute optogenetic inhibition, TOX-mediated AHN GABA cell inhibition achieves irreversible and permanent dysregulation of stress-induced HPA activity. Evidence from both human and animal studies suggest that a long-term exposure to chronic dysregulation of stress response increases risk for affective and cognitive impairments. To this end, we examined TOX and GFP control mice in a battery of behavioral tests that assess anxiety-related behaviors and recognition memory for object and social cue after 4 weeks of AAV infusion and TOX expression.

**Figure 5:**
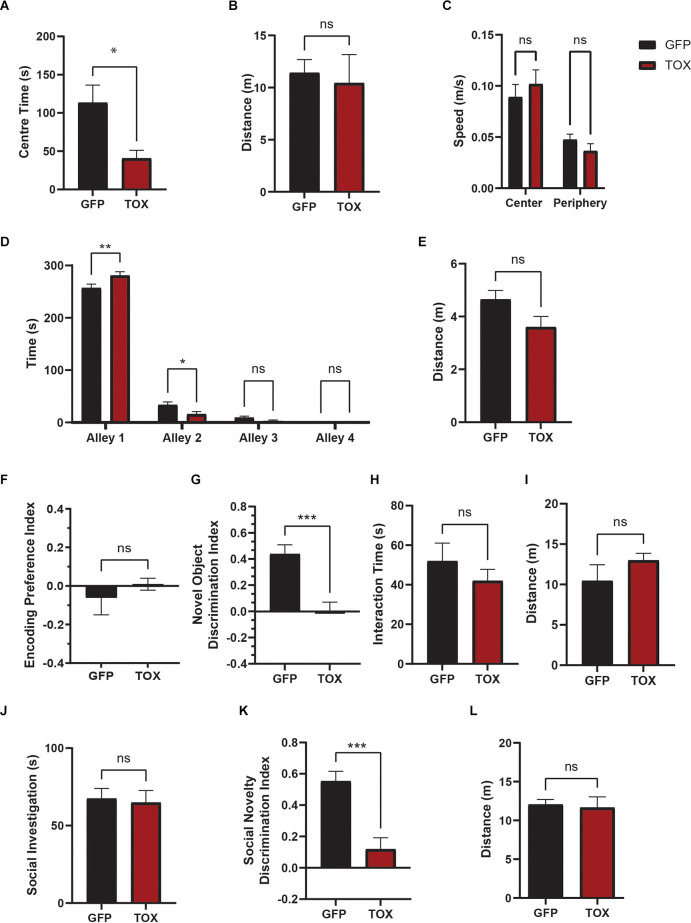
AHN GABA synaptic silencing results to abnormalities in anxiety behavior and cognitive impairments, affective and cognitive functions.** (A)** TOX mice are more anxious and avoid center area of the open field (OF) (GFP *N* = 13, TOX *N* = 10, unpaired *t*-test, *t* = 2.595, df = 21, *p = 0.0169). **(B)** Total distance traveled in open field did not differ between TOX and GFP (unpaired *t*-test, *t* = 0.3521, df = 21, *p* = 0.7283, NS). **(C)** Mean speed (m/s) of centerand periphery zone did not differ between TOX and GFP (2-WAY ANOVA, Zone × AHN treatment, *F*_(1,42)_ = 1.337, *p* = 0.2541, NS, zone effect, *F*_(1,42)_ = 26.97, ****p < 0.0001, AHN treatment effect, *F*_(1,42)_ = 0.007982, *p* = 0.9292, NS, Bonferroni’s multiple comparisons test, CenterTOX vs. GFP, *p* = 0.7669, NS, Periphery ArchT vs. GFP, *p* = 0.9096, NS). **(D)** TOX mice prefer closed alley (alley 1) and avoid open alleys (alley 2–4) compared to GFP (GFP *N* = 13, TOX *N* = 12, 2-WAY ANOVA, Alley × AHN treatment, *F*_(3,92)_ = 7.027, ***p = 0.0003, Alley effect, *F*_(3,92)_ = 1612, ****p < 0.0001, AHN treatment effect, *F*_(1,92)_ = 0.1289, *p* = 0.7204, NS, Bonferroni’s multiple comparisons test, Alley 1 GFP vs. TOX, **p = 0.0015, Alley 2 GFP vs. TOX, *p = 0.0293, Alley 3 GFP vs. TOX, *p* = 0.9960, NS, Alley 4 GFP vs. TOX, *p* > 0.9999, NS). **(E)** Total distance traveled in successive alleys did not differ between TOX and GFP (unpaired *t*-test, *t* = 2.007, df = 23, *p* = 0.0566, NS). **(F)** During the encoding phase, both TOX and GFP mice showed little preference bias between two identical objects (GFP *N* = 8, TOX *N* = 9, unpaired *t*-test, *t* = 0.7744, df = 15, *p* = 0.4508, NS). **(G)** TOX mice displayed significant impairment in novel object (NO) discrimination compared to the GFP mice (unpaired *t*-test, *t* = 4.465, df = 15, ***p = 0.0005). **(H)** Total interaction time during novel object discrimination did not differ between GFP and TOX (unpaired *t*-test, *t* = 0.9489, df = 15, *p* = 0.3577, NS). **(I)** Distance traveled during novel object discrimination did not differ between GFP and TOX (unpaired *t*-test, *t* = 1.22, df = 15, *p* = 0.2413, NS). **(J)** TOX and GFP mice investigate a stranger 1 mouse equal amount of time (GFP *N* = 11, TOX *N* = 8, unpaired *t*-test, *t* = 0.2497, df = 17, *p* = 0.8058, NS). **(K)** TOX mice displayed impaired social novelty discrimination compared to GFP (unpaired *t*-test, *t* = 4.470, df = 17, ***p = 0.0003). **(L)** Distance traveled during the social novelty discrimination did not differ between TOX and GFP mice (unpaired *t*-test, *t* = 0.2768, df = 17, *p* = 0.7853, NS). All results reported are mean ± s.e.m. *p < 0.05, **p < 0.01, ***p < 0.001, ****p < 0.0001.

TOX mice displayed significantly greater avoidance of the center of open field, indicative of increased anxiety (Figure 5A). The total distance traveled, mean speed in center or periphery zone did not differ between groups (Figures 5B,C). Consistently, TOX mice also spent more time in the enclosed alley 1 in the SUA test compared to GFP mice (Figure 5D). In both assays the total distance traveled did not differ between groups, indicating that the increased anxiety was not due to change in overall locomotor behavior (Figures 5B,E). To test short-term recognition memory, we first leveraged a novel object recognition task with a 30 min delay. During the encoding phase, both TOX and GFP control mice explored two different novel objects with equal preference (Figure 5F). After a 30 min delay, mice were reintroduced to the previously explored object and a novel object. GFP control mice spent significantly greater time exploring the novel object as detected by the novel object discrimination index (Figure 5G). However, TOX mice displayed no preference for the novel object, indicating an impaired ability to recognize familiar objects (Figure 5G). Object interaction time and total distance traveled did not differ significantly between the two groups (Figures 5H,I). Next, we asked whether the memory deficit in object recognition in TOX mice also extends to social recognition memory. Mice have an innate preference for social novelty when introduced to familiar vs. novel conspecifics, and disruptions of social behaviors and social recognition are thought to be characteristic of a variety of neuropsychiatric disorders such as depression, autism spectrum disorders, bipolar disorders, and schizophrenia. During the first sociability phase of the three-chamber social interaction test, both control and TOX mice showed a greater preference for the social chamber investigating the stranger mouse (stranger 1; Figure 5J) and spent less time investigating the empty chamber (data not shown). After a 3 min delay, a second new stranger mouse (stranger 2) was placed in the empty chamber for a 10 min social novelty recognition. GFP control mice spent significantly greater time interacting with the new stranger two mouse than the familiar stranger one mouse as detected by the discrimination index (Figure 5K). However, TOX mice displayed no preference for the stranger two mouse, indicating an impaired ability to recognize familiar social cues (Figure 5K) without change in locomotion (Figure 5L).

Together, these findings suggest that irreversible and permanent inhibition of AHN GABA cells in TOX mice leads to an increase in anxiety-related behavior and impaired recognition memory.

## Discussion

The present study extends previous findings that the vHPC suppresses neuroendocrine responses (e.g., HPA activity) to psychogenic stressors (Herman et al., 1998; Radley and Sawchenko, 2011). We first demonstrated that the vHPC are monosynaptically connected to GABA cells in the AHN. Next, using pathway-specific optogenetic inhibition, we showed a role for the HPC→AHN pathway in negative feedback inhibition of the HPA axis. Inhibition of the pathway during a physical restraint stress resulted in a prolonged release of CORT after stress and an increase in anxiety-related behaviors. Importantly, direct inhibition of AHN-GABA cells produced the same effects on stress-induced neuroendocrine responses, corroborating the role of HPC→AHN pathway in mediating the hippocampal control of stress responses. Finally, we found chronic inhibition of AHN GABA cells results in increased anxiety-related behavior and cognitive impairment.

### A Distinct Connectivity of vHPC and Its Relevance to Stress Responses

It is well-established that there is a dorsoventral segregation of neural connectivity and function within the hippocampus (Herman et al., 1998). The dorsal hippocampus (dHPC) is heavily connected to the associational cortical regions including the perirhinal, postrhinal, and retrosplenial cortex, whereas the ventral hippocampus (vHPC) projects to the prefrontal cortex as well as various subcortical regions implicated in stress, emotion and affect, including the BNST, lateral septum, amygdala, and hypothalamus. In keeping with their distinct connectivity patterns, numerous studies have shown that the dHPC plays a major role in the processing of spatial and mnemonic information whereas the vHPC plays a particular role in emotional processing (Richmond et al., 1999; Bannerman et al., 2002, 2003, 2004; Kjelstrup et al., 2002).

### Activation of Hippocampal-Hypothalamic Circuit During Stress Responses

When an animal is exposed to acute stress such as physical restraint or foot shock, the amygdala and brainstem catecholamine pathways are activated, sending excitatory drives to the parvocellular PVN neurons (Roozendaal et al., 2007). The PVN neurons in turn secrete a cocktail of adrenocorticotropic hormone (ACTH) secretagogues including CRF and arginine-vasopressin (AVP) into the portal vasculature that supplies the anterior pituitary (Antoni, 1986; Roozendaal et al., 2007; Herman et al., 2016). The subsequent release of ACTH from the anterior pituitary then elicits the synthesis and secretion of glucocorticoids by the adrenal cortex, initiating the physiological responses to stress. In parallel, multimodal sensory information regarding environmental context is encoded by a distinct neural ensemble within the hippocampus, forming a short-term contextual memory of the stress event. This information is later sent by the dHPC to various parts of the neocortex for long-term memory formation, and the hippocampus-dependent memory of stressful events is later retrieved when animals encounter stress-associated context (Nyberg et al., 2000; Tanaka et al., 2014).

In addition to stress-associated contexts, the hippocampus receives endocrine feedback signals about the level of glucocorticoids released from the adrenal gland following initial exposure to a stressor. The potential for the hippocampal neurons to tune stress responses is highlighted by its rich expression of glucocorticoid (GR) and mineralocorticoid (MR) receptors. Notably, the hippocampus displays the differential distribution of GR and MR along its dorsoventral axis. The low-affinity GR has been found to be more abundant in the dHPC while the vHPC has a higher prevalence of high-affinity MR (Robertson et al., 2005). Furthermore, hippocampal modulation by GR and MR appears to be biphasic; the MR activity promotes the excitability of the hippocampal network, whereas the GR seems to suppress network activity (Maggio and Segal, 2007). This observation led to a hypothesis that acute stress may produce opposite effects on the dHPC and vHPC network, switching from the dHPC dominant state to the vHPC dominant state (Maggio and Segal, 2009; Segal et al., 2010). In the normal (non-stressful) state, the dHPC network is kept highly excitable. During and after acute stress, however, the dHPC network becomes less excitable, and the vHPC and its connections to subcortical regions are strengthened, dominating the overall hippocampal information flow. Although it needs to be experimentally determined, we speculate that the AHN-projecting vHPC neurons likely express greater levels of MR than GR.

### Hippocampal-Hypothalamic Circuit Mediates Negative Feedback Inhibition of Stress Responses

Both HPC→AHN pathway inhibition (by optogenetic approach) and AHN GABA cell inhibition (by TOX) produced a remarkably similar effect on CORT profile during and after restraint stress in adult male mice. While the baseline CORT level did not differ from control at the beginning of the restraint stress, both ArchT and TOX mice displayed a significantly blunted initial CORT response during restraint stress and a delayed CORT peak at 60 min compared to a peak at 30 min in controls. Even at the 120 min time point (i.e., 90 min after stress was terminated), CORT levels in ArchT and TOX mice remained as high as they were at the peak. These results suggest that inhibiting vHPC inputs to AHN-GABA cells impaired negative feedback inhibition of the HPA axis. The mechanism underlying this effect, however, remains unknown. The most plausible interpretation is that AHN GABA cell inhibition caused a slow rise of CORT during the restraint, which then led to a decrease in rate-sensitive negative feedback and a delayed termination of the CORT response. The finding that AHN GABA cell inhibition decreased CORT level during restraint stress is unexpected and contradictory to the concept that AHN GABA cells provide direct inhibitory inputs to the CRF-expressing parvocellular neurons in the PVN. Future studies will need to examine the firing frequency of CRF neurons in the PVN while activating or inhibiting vHPC axon terminals in the AHN in* ex vivo* slice recordings.

There are several factors to consider in interpreting our findings. First, the present study did not determine whether HPC→AHN pathway inhibition or AHN GABA cell inhibition alters anxiety behaviors in non-stressed animals (i.e., a baseline anxiety level). Therefore, it is plausible to suggest that HPC→AHN pathway inhibition or AHN GABA cell inhibition may produce a long-lasting increase in baseline anxiety, regardless of subsequent exposures to physical stress. Importantly, however, our recent study measured the effect of optogenetic HPC→AHN pathway inhibition on anxiety behaviors in non-stressed animals using three different paradigms: open field, elevated plus maze, and successive alleys, and showed that HPC→AHN pathway inhibition does not affect baseline anxiety (Bang et al., 2022). Next, stress response and anxiety behaviors differ between sexes. We examined stress-induced CORT response in a separate cohort of AHN GABA-TOX female mice (data not shown) and detected a similar delayed profile of circulating CORT. In addition, AHN GABA-TOX females displayed significant cognitive impairments in novel objects and social novelty discrimination compared to GFP control females. Of note, however, we did not observe a significant change in anxiety behaviors in AHN GABA-TOX females, which may be due to a lack of control for the estrus cycle stage in the females in our experiment design.

### Contribution of the LS-AHN and mPFC-BNST Pathways in Controlling Stress Responses

While our finding supports the idea that the vHPC plays a critical role in negative feedback regulation of HPA axis activity, it does not rule out the contribution of other multisynaptic limbic inputs to the PVN in attenuating neuroendocrine responses. A recent study by Anthony et al. demonstrated the contribution of the LS-AHN pathway in regulating the HPA axis and stress-induced anxiety behaviors (Anthony et al., 2014). Using *in vivo* optogenetic approach, the study demonstrated that LS-GABA neurons expressing CRFR2 form inhibitory synapses on the PVN-projecting GABA neurons in the AHN, and that stimulating the LS-AHN pathway increased CORT level and stress-induced anxiety behaviors after acute physical restraint stress. This suggests that the LS-AHN pathway disinhibits the HPA axis activity further promoting anxiety behaviors. It is plausible that the excitatory vHPC inputs and inhibitory LS inputs converge on AHN GABA cells during an acute stress response. According to this model, the dynamic balance between excitatory and inhibitory inputs arriving at the AHN from two limbic structures may determine the excitability of AHN GABA cells and their inhibitory inputs to the PVN. Electrophysiology experiments focusing on AHN GABA cells should be able to determine this possibility.

Similarly, mPFC inputs to the BNST have been implicated in top-down inhibition of the HPA axis response; mPFC lesions increase HPA secretory responses to stress (Diorio et al., 1993; Radley et al., 2006) while corticosterone infusion into the mPFC attenuates stress-induced HPA activation (Diorio et al., 1993). Radley et al localized the HPA-inhibitory influences of the mPFC to the dorsal area of mPFC containing largely the prelimbic region (Radley et al., 2006). The study compared the effects of lesions of the prelimbic and infralimbic mPFC regions on acute restraint stress-induced c-Fos activation in the PVN. Stress-induced c-Fos response in the PVN as well as the serum level of ACTH and CORT were markedly exaggerated after lesions of the prelimbic, but not the infralimbic area, suggesting that the prelimbic area of the mPFC exerts inhibitory influences on neuroendocrine outputs in response to stress. Subsequent works from the same group showed that prelimbic lesion reduced c-Fos responses to acute restraint stress in PVN-projecting GABA neurons in the BNST, indicating that the BNST is a potential GABAergic relay structure mediating the prelimbic inhibition of HPA responses (Radley et al., 2009). Importantly, it was later demonstrated that vHPC inputs and mPFC inputs converge onto the same PVN-projecting GABA neurons in the BNST, suggesting that limbic inputs arising from the vHPC and mPFC to BNST coordinate to inhibit the stress responses (Radley and Sawchenko, 2011). A question of interest, therefore, is how the AHN-projecting vHPC cells are anatomically and functionally related to the BNST-projecting vHPC cells. A plausible scenario is a considerable overlap between the two cell populations where the same group of vHPC cells send collateralized axons to both BNST and AHN. Activation of such overlapping cell populations would activate both AHN and BNST simultaneously and exert an additive influence on the PVN activity and stress responses.

## Data Availability Statement

The original contributions presented in the study are included in the article, further inquiries can be directed to the corresponding author.

## Ethics Statement

The animal study was reviewed and approved by University of Toronto, LACC.

## Author Contributions

JB and JK contributed to design of the study and manuscript drafting. JB, JZ, MR, and SS-C performed experiments. JB, JK, and PM contributed to conceptual design. All authors contributed to the article and approved the submitted version.

## Conflict of Interest

The authors declare that the research was conducted in the absence of any commercial or financial relationships that could be construed as a potential conflict of interest.

## Publisher’s Note

All claims expressed in this article are solely those of the authors and do not necessarily represent those of their affiliated organizations, or those of the publisher, the editors and the reviewers. Any product that may be evaluated in this article, or claim that may be made by its manufacturer, is not guaranteed or endorsed by the publisher.
